# From Research Question to Conducting a Randomized Controlled Trial on Continuous Antibiotic Prophylaxis in Prenatal Hydronephrosis: A Rational Stepwise Process

**DOI:** 10.3389/fped.2016.00027

**Published:** 2016-03-30

**Authors:** Luis H. Braga, Bethany Easterbrook, Kizanee Jegatheeswaran, Armando J. Lorenzo

**Affiliations:** ^1^Division of Urology, McMaster University, Hamilton, ON, Canada; ^2^Department of Clinical Epidemiology and Biostatistics, McMaster University, Hamilton, ON, Canada; ^3^Department of Pediatrics, McMaster University, Hamilton, ON, Canada; ^4^McMaster Pediatric Surgery Research Collaborative, McMaster University, Hamilton, ON, Canada; ^5^Division of Urology, The Hospital for Sick Children, Toronto, ON, Canada

**Keywords:** hydronephrosis, urinary tract infections, antibiotic prophylaxis, prenatal, children, randomized controlled trials, pilot study

## Abstract

**Introduction and objectives:**

Continuous antibiotic prophylaxis (CAP) use to prevent urinary tract infections (UTI) in infants with prenatal hydronephrosis (HN) remains controversial. Lack of consensus guidelines and diverse practice patterns for postnatal management of HN highlight the dire need for higher level of evidence studies. Herein, we aim to describe the steps from developing a well-defined research question to execute a multicentered randomized controlled trial (RCT) to address the issue of CAP use in patients with prenatal HN.

**Materials and methods:**

The steps involved were (1) choosing the proper research question, (2) survey of practice patterns and establishing clinical equipoise, (3) systematic review of the literature, (4) reviewing own practice, (5) longitudinal prospective study, (6) pilot study, (7) cost-utility analysis, and (8) definitive RCT (clinical trials registry number: NCT01140516). An update of our previous systematic review was conducted using two electronic databases and gray literature from 2010 to 2015. Eligibility criteria included studies of children <2 years old with postnatally confirmed prenatal HN, receiving CAP or not, and reporting on development of UTIs, capturing information on voiding cystourethrogram result and HN grade. Full-text screening was conducted by two independent reviewers. UTI rates in patients with high-grade HN were compared across different study designs. Finally, blinded comparative analysis of UTI rates between placebo and treatment groups was carried out using chi-square test.

**Results:**

UTI rates in patients with high-grade HN by their respective study design were: 25% for systematic review, 20% for retrospective study, 21% for prospective and pilot studies, and 13% for the definitive RCT thus far. Regardless of the type of study design, patients with hydroureteronephrosis had significantly higher (threefold to sixfold) UTI rates than those with isolated HN. Our updated systematic review yielded 486 citations, of which 9 (*n* = 1987 infants) observational studies met eligibility criteria.

**Conclusion:**

UTI rates in patients with high-grade HN dropped from 25% in observational studies to 13% in our RCT. This decline in UTI rate demonstrates that study designs lacking strategies to minimize bias are more prone to overestimate treatment effects. These findings highlight the importance of conducting methodologically sound RCTs to answer clinically meaningful questions, such as the one presented here.

## Introduction

*In utero* dilation of the renal collecting system, commonly called prenatal or antenatal hydronephrosis (HN), is one of the most commonly diagnosed congenital abnormalities, detected in up to 5% of all pregnancies (1). Physiologic- (i.e., transient) isolated HN (so called “ureteropelvic junction obstruction-like” or UPJO-like) and vesicoureteral reflux (VUR) are the most frequent etiologies, followed by non-refluxing primary megaureter [hydroureteronephrosis (HUN)] (1). Infants born with prenatal HN have been shown to have a 12-fold higher risk of hospitalization for urinary tract infections (UTI), predominantly in the first year of life (2). Children with Society of Fetal Urology (SFU) grades III–IV HN are at a particularly increased risk, having a much higher incidence of UTI when compared to those with lower HN grades (3–6).

The potential benefit of continuous antibiotic prophylaxis (CAP) in preventing UTIs is often weighted against the perceived modest impact of this intervention, along with concerns of developing bacterial antibiotic resistance and other theoretical long-term adverse effects. As a result, the decision to recommend CAP for patients with prenatal HN remains controversial. The American Urological Association (AUA), the SFU, and the Canadian Urological Association (CUA) all acknowledge that use of CAP for UTI prevention in infants with prenatal HN has been based on low level of evidence. Consequently, in 2009, the CUA guidelines on prenatal HN provided grade D recommendation for CAP use in children with this condition (7). Not surprisingly, this lack of high-quality evidence has resulted in multiple guidelines with varying criteria for prescribing CAP. In 2010, the SFU Consensus Statement on HN recommended prophylaxis only for infants with high-grade HN and those with VUR (8). On the other hand, the AUA guidelines suggested the use of CAP for children with asymptomatic VUR (i.e., without previous history of UTI) to be optional (9). Given the uncertainty regarding CAP use in prenatal HN patients, an effort to identify knowledge gaps in the literature and find proper evidence-based recommendations is well-timed and certainly needed.

As a result, the objective of this review is to describe the steps necessary to take an investigator from developing a research question to executing a large multicenter randomized controlled trial (RCT) aimed at addressing the issue of CAP in newborns and infants with prenatal HN.

## Step 1 – Choosing the Proper Research Question

Interest in a particular topic usually triggers the research process, but it is the in-depth familiarity with the subject that helps define a certain aspect of care for a study. Questions usually arise out of perceived knowledge deficits within that topic. It is important to know “where the boundary between current knowledge and ignorance lies” (10). To exemplify this concept, Donald Rumsfeld coined the term *known* “*knowns*,” to highlight things we feel are well acknowledged and accepted. For example, we can all agree that some children with prenatal HN are at risk and may indeed develop febrile UTIs (fUTI). There are also *known* “*unknowns*,” which refer to things we admit as not known (11). We are uncertain whether CAP reduces the risk of UTIs in patients with prenatal HN (although some may dispute it, based on limited data). Having such a structured and curious mindset to find gaps in the literature is essential to developing a proper research question.

A research question worth pursuing should encompass the following aspects, captured in the acronym *FINER* (10): it should be *F*easible, i.e., related to a condition that affects an important number of patients, and thus, can be subjected to scrutiny with a reasonable chance of reaching adequate sample size; be *I*nteresting to the scientific community and to the public; be *N*ovel, as granting agencies are unlikely to fund projects that aim at mirroring similar previous studies; be *E*thical, an undebatable requirement that rightfully obeys the strict guidelines set by research ethics boards and declarations on ethical conduction of trials (12); and finally, be *R*elevant to an extent that it will favorably impact clinical practice. Prenatal HN fits all these criteria, particularly in terms of clinical equipoise and lack of properly conducted randomized placebo controlled trials reported in the literature.

## Step 2 – Survey of Practice Patterns and Establishing Clinical Equipoise

Clinical equipoise refers to genuine uncertainty among clinicians regarding the value or superiority of a therapeutic strategy or medical intervention. The clinical dilemma regarding management of prenatal HN has been confirmed by recent surveys, all showing that the decision to prescribe CAP is heavily influenced by provider factors, such as length of years in practice, geographic location, specialty, along with the severity of upper tract dilation. For example, pediatric urologists in practice for more than 15 years are less likely to initiate CAP at birth than younger practitioners (<15 years) (13). By geographic region, American physicians are twice as likely to prescribe CAP when compared to their European colleagues, whose prescribing decision was based mainly on the prenatal renal pelvis diameter (13). According to a French-speaking survey, 23% of pediatric nephrologists and 31% of pediatric urologists would start CAP immediately after birth (8). In contrast, 56% of general pediatricians would routinely prescribe CAP for infants with prenatal HN (4). By specialty, nephrologists were more likely to prescribe CAP for bilateral low-grade HN compared to pediatric urologists (14). The former group was also more likely to prescribe CAP for high-grade “UPJO-like” HN. Across Canada, only 20% of participating institutions reported following standardized protocols for postnatal management of prenatal HN (14). Similarly, Zanetta et al. also documented a wide variation in the use of CAP and indication for voiding cystourethrograms (VCUG) among American pediatric urologists, radiologists, and obstetricians. According to their survey, agreement on duration of follow-up for mild prenatal HN is missing (15).

This lack of consensus, leading to different management strategies for prenatal HN, coupled with paucity of high-level, evidence-based guidelines, compels physicians to rely solely on their clinical experience and judgment. As a result, controversy is evident among the medical community surrounding the use of CAP for infants with prenatal HN, clearly exemplifying the principle (and presence) of clinical equipoise.

## Step 3 – Critical Appraisal of the Literature

Lack of consensus guidelines and diverse practice patterns for postnatal management of prenatal HN undoubtedly derives from conflicting and inconsistent data in the literature. Given these discrepancies, we previously published a systematic review with the goal of summarizing the latest evidence regarding CAP use in prenatal HN patients (5). Thus far, it remains the only published meta-analysis focusing on this topic, reviewing 1681 titles and abstracts, of which 21 full-text articles including data from nearly 4000 patients were extracted and analyzed (3, 5, 6, 16–34). Pooled UTI rates were six times higher for high-grade HN patients when compared to those with low-grade HN, similarly to Lee et al.’s study, which showed that the UTI rate rose from 12% in children with grade II HN to 40% in those with grade IV (6). UTI rates were equivalent in children with low-grade HN regardless of their CAP status (2.2% on CAP vs. 2.8% not on CAP, *p* = 0.51). On the contrary, high-grade HN patients on CAP experienced fewer UTIs than those not on antibiotics (14.6 vs. 28.9%, *p* < 0.01), suggesting that CAP may be beneficial for this patient population. The estimated number needed to treat (NNT) was 7, meaning that a clinician must offer CAP to 7 patients with high-grade HN in order to prevent 1 UTI (5).

As it is the case with any systematic review, the applicability of this meta-analysis heavily depends on the quality and validity of the included studies (35). The review’s main limitation was the inclusion of low- to moderate-quality (76%) observational studies (5). Despite the lack of high-quality RCTs on the use of CAP for prenatal HN, many clinicians are likely to be influenced or change clinical practice based on observational studies alone, which may not be ideal. In addition, heterogeneous and inconsistent grading of HN severity across studies limited the ability to compare UTI rates from all included studies. Lastly, the association between UTI and important confounding variables, such as presence of VUR, gender, and circumcision status, could not be investigated due to the paucity of reported data. Nevertheless, despite these limitations, this systematic review has reflected the most comprehensive review of the literature on the use of CAP for prevention of UTIs in children with prenatal HN thus far and sets the stage for further exploring the topic.

In order to update the previous systematic review on prenatal HN with studies published from 2010 to 2015, we conducted a comprehensive search of EMBASE and MEDLINE databases, including key search MeSH terms to reflect the following subjects: (1) HN, (2) prenatal, (3) antibiotics prophylaxis, and (4) UTI. Articles were screened in duplicate based on the following eligibility criteria: (1) primary diagnosis of prenatal HN, (2) all subjects aged <2 years, (3) intervention arms included CAP, no treatment, or both, (4) reported rate of UTI, (5) reported number of patient who underwent VCUG, and (6) HN grade according to the SFU classification and/or anteroposterior diameter (APD) of the renal pelvis. Case reports, case series with <10 subjects, and review articles were excluded. After screening the title and abstracts of 486 hits, 39 citations were reviewed in full-text and their references were hand searched. Of the 39 full-texts reviewed, 9 observational studies met all eligibility criteria (36–45).

Szymanski et al., studying 206 consecutive children with postnatally confirmed prenatal HN, reported that patients with high-grade HN had a threefold greater risk of UTIs vs. those with low-grade, after adjusting for gender and circumcision status. Children with high-grade HN had 11.1 infections per 100 patient-years compared to 3.52 infections per 100 patient-years in those with low-grade HN (*p* = 0.02) (36). Gender, circumcision status, and presence of VUR (all patients with reflux were on CAP) were not found to be independent risk factors for UTI (36), analysis that may have been limited by the few number of UTI events and the retrospective design of their study.

Islek et al. studied a specific group of 84 prenatal HN patients with UPJO-like (isolated HN) of different HN grades. The authors concluded that CAP did not change the risk of UTI, regardless of HN severity, as none of their 84 patients with UPJO-like type of HN developed UTI within a median follow-up period of 18 months (37). Due to the lack of UTI data segregated by HN etiology (stratification of UTI rates by isolated HN vs. HUN) in most of the included studies in Braga et al.’s systematic review, it was not possible to comment on the distinct association between UPJO-like dilation and UTI risk, as pointed out by Islek et al. (37). Nonetheless, the systematic review did suggest that the pooled rate of UTI in patients with HUN was significantly higher than that of all children with high-grade HN (34 vs. 23%, *p* < 0.05). Gimpel et al. corroborated these findings by noting an overall 45% UTI rate in 49 patients with 56 primary obstructive megaureters. These authors observed a 50% absolute risk reduction in UTI rate of children on CAP compared to those not receiving prophylaxis (94 vs. 42%, *p* < 0.01) (38).

Herz et al. observed a 22% UTI rate in 405 patients with prenatal HN. These authors found a significantly lower UTI rate in children receiving CAP compared to those not on antibiotics (7.9 vs. 18.7%, *p* = 0.02). In addition, based on their multivariate analysis, presence of ureteral dilation, high-grade VUR, and UVJ obstruction (primary megaureter) were independent risk factors for development of UTI (39). More specifically, children with ureteral dilation >11 mm not receiving CAP had a 5.54 (OR = 5.54; CI = 3.15–7.42, *p* = 0.001)-fold increased risk of fUTI compared to those on CAP (39).

A study by Duzenli et al. captured by our updated gray literature search, showed a 22% UTI rate in infants with postnatally confirmed prenatal HN, even though only 36% of the entire group of 136 patients were on CAP (40).

Sencan et al. conducted a prospective review of 760 patients with mild prenatal HN, defined in the study as an anterior–posterior pelvic diameter of 7–10 mm. Of the 692 patients who had complete clinical data, 23 (3.3%) developed a UTI at mean and median ages of 7.4 and 3 months, respectively. Eighteen (78.3%) of the 23 patients underwent a VCUG and two (8.7%) had high-grade VUR. Uncircumcised males had a 7.8 times greater risk of developing UTI compared to those who were circumcised (*p* < 0.001) (41).

A prospective observational study conducted by Di Renzo et al. examined 47 children with 58 primary non-refluxing megaureters and found an overall rate of fUTI of 15%, with 6 (13%) of these patients requiring hospitalization. The majority of fUTIs developed after 6 months of age, contradictory to much of the current literature (42).

A recent study by Zee et al. examined patients prospectively enrolled in the SFU HN registry to determine incidence and factors associated with UTI in children with prenatal HN. Two hundred and thirteen patients (154 males) from four medical centers were enrolled, with 9% of patients developing a UTI. Mulitvariate analysis identified females (OR 7.3, CI 2.2–22, *p* < 0.001), uncircumcised males (*p* < 0.01), and presence of renal cyst (*p* < 0.05) as significant risk factors for UTI. Interestingly, this cohort provided no evidence of protective effect regarding CAP administration in patients with HN (43).

Finally, in a prospective review of 80 patients with primary non-refluxing megaureter, Braga et al. observed a 34% fUTI rate. Using a Cox regression analysis, they found that uncircumcised males (HR = 3.4, 95% CI: 1.1–10.7, *p* = 0.04) and lack of CAP (HR = 4.1, 95% CI: 1.3–12.7, *p* = 0.01) were independent risk factors for the development of fUTI in this subgroup of prenatal HN patients (44). Similarly, a recent review article on HN identified circumcision as having a protective effect in preventing UTIs, and hence, suggested that either performing elective circumcision or prescription of CAP be considered as part of the postnatal management plan for prenatal HN patients (45).

## Step 4 – Reviewing Your Own Practice

Given the paucity of data on UTI risk factors in the HN literature and the inability of our systematic review to provide strong evidence regarding the association between patient characteristics and UTI, we sought to review the postnatal management of children with prenatal HN at our own institution, aiming to specifically explore the following *a priori* identified variables: HN grade, gender, circumcision status, HN etiology/type, use of CAP, and VUR status (46). Following institutional ethics approval (10-594-C), this study reviewed available data on 376 patients, 99 females and 277 males, with postnatally confirmed prenatal HN who were seen in a single pediatric urology outpatient clinic. Even though retrospective in nature, we sought to improve the accuracy of diagnosing UTIs by only including events that had a positive urine culture with >100000 cfu of a single microorganism in a catheterized specimen and associated with temperature ≥38°C. CAP was prescribed more often to female infants vs. males (71 vs. 57%, *p* < 0.01), those with high- vs. low-grade HN (70 vs. 55%, *p* < 0.01), and to children with VUR vs. those without reflux (96 vs. 51%, *p* < 0.01). Circumcision status was not observed to play a role in changing CAP prescribing patterns (47).

Similarly to others (5, 36), we confirmed that patients with high-grade HN were at a higher risk of UTI when compared to those with low-grade (adjusted OR 2.4, 95% CI: 1.3–4.6). In addition, this study was the first to clearly demonstrate that circumcision status is an important confounder of the association between gender and UTI risk in this patient population. The risk of UTI was three times higher in females (adjusted OR: 3.2; 95% CI: 1.0–10.2) and uncircumcised males (adjusted OR: 3.6; 95% CI: 1.2–11.2) compared to circumcised boys. As such, the relative risk of UTI in female vs. male infants in any HN population is bound to depend on the proportion of males who are circumcised and simply segregating by gender without consideration of this important covariate is bound to make analyses incomplete and potentially erroneous (46). Three other studies shared similar findings, asserting that the foreskin on uncircumcised males and external genitalia in baby girls harbors pathogenic bacteria, explaining the increased UTI risk (2, 3, 47).

In addition, we also observed a threefold higher UTI rate in patients with HUN compared to those with isolated HN (i.e., “UPJO-like”) (23 vs. 7%, *p* = 0.03). These results were supported by at least two previous studies, which hypothesized that urinary stasis in a dilated collecting system or tortuous ureter would facilitate bacteria colonization and growth, providing a conceptual basis for infection risk (6, 33). Interestingly, contrary to the systematic review results, our retrospective study found no association between CAP use and UTI rates. Such finding, which differs from prior studies reporting benefit in UTI prevention (39), exemplifies uncertainty over the real efficacy of this prophylactic intervention in patients with prenatal HN. The main limitation of our retrospective study was the inability to obtain detailed information on all patients. For example, compliance to CAP could not be assessed. If CAP truly impacted UTI rates, then non-compliance would lead to a reduced benefit margin between groups (type II error), even though a true difference exists. Not adjusting for such a confounder may have biased the results, changing a treatment effect from positive to negative or no effect. This highlights the importance of adjusting for clinically relevant confounders with more stringent research strategies (13–15).

## Step 5 – Longitudinal Prospective Study

Learning from the shortcomings of our previous analyses, we sought to verify the impact of gender, circumcision status, HN grade, VUR status, type of HN, and CAP use on rate and time to UTI in a prospective fashion (48). By doing so, we confirmed that females and uncircumcised males had a significantly higher UTI risk compared to that of circumcised males (HR for females = 3.4, 95% CI: 1.2–9.2; HR for uncircumcised males = 3.2, 95% CI: 1.2–8.4; *p* = 0.02). Likewise, Herndon et al. have shown that uncircumcised males experienced a fourfold higher risk of UTI compared to circumcised boys with prenatal HN (19). In addition, as indicated in the systematic review (5) and in a recent study by Herz et al. (39), we also found that CAP reduced the risk of UTIs in children with postnatally confirmed prenatal HN when compared to those not receiving antibiotics (HR = 5.2; 95% CI: 2.7–10.0; *p* < 0.01).

Although prospective, this study was observational (ethics approval: 13-162-D). Thus, recommendation to obtain a VCUG in our cohort was based on physicians’ discretion, practice that is variable considering that its value remains controversial (19, 20, 33, 49). Nevertheless, we documented that diagnosis of VUR was strongly associated with development of UTIs in this population (HR = 20.8; 95% CI: 10.2–42.3; *p* < 0.01). This is clinically relevant as it establishes VUR as an important risk factor for UTI, not only for children diagnosed following a first episode of fUTI (50) but also in asymptomatic patients identified solely due to the presence of prenatal HN. While some authors caution the indication of VCUG in all children after development of the first fUTI (51), others have reported that a VCUG can be safely performed in high-grade HN patients without increasing the risk of UTI (52).

As it occurs with any observational study, limitations innate to the nature of the study design do not allow controlling for unknown confounding variables, which may lead to an overestimation of the overall UTI rate. Therefore, as a logical next step to consolidate the evidence regarding effectiveness of CAP in reducing UTIs in children with prenatal HN, we decided to conduct a RCT comparing prophylactic antibiotic to placebo in this population.

## Step 6 – Designing a Feasibility Pilot Study

Randomized controlled trials are considered as the gold standard for evaluating the effectiveness of an intervention, thus ranking among the highest levels of evidence when properly conducted. However, doing an RCT that involves vulnerable groups, such as children, poses an enormous recruitment challenge. Difficulties in recruiting and guaranteeing compliance for a large planned sample of patients often demands expanding the number of study sites, which adds to costs for coordination. With the ultimate goal of conducting a large multicenter RCT, we sought to first assess its feasibility. Thus, our aim was to conduct a pilot trial on the effect of CAP on fUTI rate in infants with high-grade HN. More specifically, our feasibility study focused on determining barriers and realistic expectations for recruitment rate, determining an event rate within the confines of an experimental study, and assessing medication compliance and optimal outcome measures.

Following institutional ethics approval (09-255) patients 1–5 months old with SFU grade III/IV isolated HN or HUN (ureteral caliber ≥7 mm), confirmed by postnatal ultrasound, and who had a VCUG to rule out VUR were included in the study. Children with low-grade HN were excluded because of their minimal risk of UTI (5) and to avoid unnecessary radiation exposure. Computer-generated, blocked randomization allocated patients on a 1:1 ratio to Trimethoprim or placebo. Participants, medical staff, research assistants and outcome assessors were blinded to the type of intervention received (53).

Of the 301 HN patients screened, 220 (73%) were not eligible to participate in the study. The most common reasons for exclusion were age at presentation >5 months (49%), low-grade HN (33%), and VUR (10%). On the other hand, of the 81 eligible children, 46 (57%) were successfully recruited, with all but 5 followed up to study completion (2 missed randomization window and 3 withdrew during the trial). Six patients (20.7%) developed fUTI over a mean follow-up period of 8.4 months. UTI was significantly more likely to occur in children with HUN than in those with isolated HN (26 vs. 4%, *p* = 0.02). Overall, high-level of compliance was demonstrated with 95% medication compliance and 98% clinic follow-up compliance rates, respectively (53).

This high adherence to study protocol provided us the confidence necessary to run a definitive trial to test the effectiveness of CAP in reducing UTIs in patients with prenatal high-grade HN. More importantly, this pilot trial was invaluable in highlighting the need to improve patient recruitment, which was achieved by extending the patient age limit to 7 months and by collaborating with other centers to create a multicenter study.

## Step 7 – Cost-Utiliy Analysis

Through the development of a probabilistic decision model, we evaluated whether prescription of CAP in infants with high-grade HN within the first 2 years of life to prevent fUTIs is an efficient use of health-care resources. The model estimated clinical outcomes, expected costs, and quality-adjusted life years (QALYs) related to two management scenarios in infants with high-grade HN: use of CAP and monitoring without CAP (54).

The base case for this analysis was set to be an infant with SFU III or IV HN, without VUR and with an APD of ≥15 mm over a 2-year time horizon. Overall, the use of CAP for prevention of UTI in infants with SFU grade III/IV HN cost CAD$1571.19 compared to CAD$1956.44 when CAP was not used. This produced an estimated cost reduction of $385.25, a decrease in outpatient UTIs by 0.21 infections, and produced 0.0001 more QALYs when compared to no CAP (54).

Overall, this probabilistic decision model provided evidence that across all three outcomes, CAP was a more efficient expenditure than no CAP for the prevention of UTI in infants with high-grade HN within the first 2 years of life.

These results were deemed promising, and when coupled with the pilot RCT data, further encouraged us to conduct the definitive RCT on use of CAP in this population. This cost-utility analysis, when combined with the results of the definitive RCT, will allow an evidence-based health policy statement regarding the effectiveness of CAP in the prevention of UTIs in infants with high-grade HN to be developed.

## Step 8 – Definitive Randomized Placebo Controlled Trial

A total of 80 participants per arm were needed to power the analysis for the definitive trial at 80%, assuming an alpha error of 5% and a conservative estimate of 20–30% event rate in the placebo group and 10% in the antibiotic group (49).

**Figure 1:**
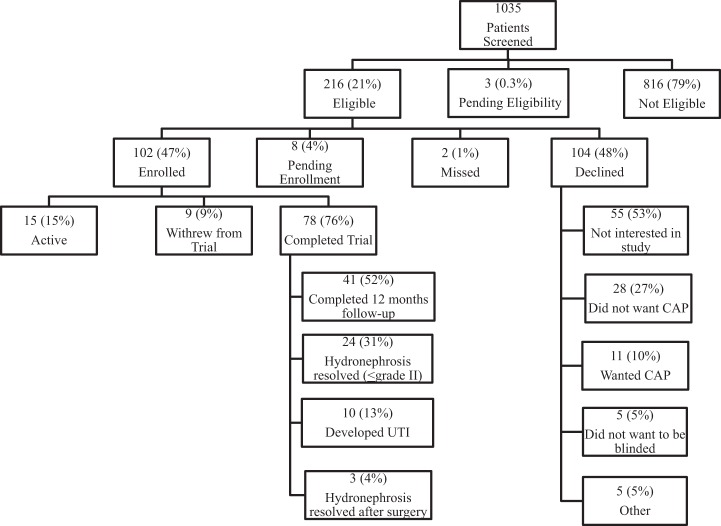
**Flow diagram of all screened patients**.

Of 1035 infants with prenatal HN screened thus far, 216 (21%) have met eligibility criteria. Of these, 104 (48%) declined to participate. Main reasons for refusal were lack of any parental interest in a research study (*n* = 55; 53%) and parental unwillingness to be blinded to the study medication (*n* = 44; 42%). Thus, ultimately 102 (47%) children were recruited (Figure 1).

Of patients enrolled in the study, 85 (83%) were males, of which, 35 were not circumcised (34%); 31 (30%) had SFU Grade IV HN, and 13 (13%) had bilateral urinary tract dilatation. Overall, 71 patients (70%) had UPJO-like and 29 (28%) primary megaureter (Table 1). Mean age at randomization was 3 months (SD = 1.7), with a median of 2.3 months (0–7 months).

**Table 1:** **Baseline demographic characteristics of enrolled patients in the definitive RCT**.

Variable	No. of patients *n* = 102 (%)
Female	17 (17)
Males	
Uncircumcised	50 (49)
Circumcised	35 (34)
Hydronephrosis side	
Lt	64 (63)
Rt	25 (24)
Bilat	13 (13)
SFU hydronephrosis grade	
III	71 (70)
IV	31 (30)
Hydronephrosis etiology	
UPJO-like (isolated hydronephrosis)	71 (70)
Primary megaureter (hydroureteronephrosis)	29 (28)
UPJ + UVJ	2 (2)
Tortuous ureter	18 (58)
Not tortuous ureter	13 (42)
Hydronephrosis management	
Conservative	79 (77)
Surgery	23 (23)
Pyeloplasty	18 (78)
Distal ureterostomy	1 (4)
Ureterocystostomy	4 (18)

Fifteen (15%) participants are still on active follow-up and 9 (9%) withdrew during the study course. Of the 78 (76%) who completed the trial, 41 (52%) completed the required 12-month follow-up without development of fUTI; 24 (31%) finished early due to resolution of HN; 3 (4%) resolved following surgery; and 10 (13%) developed the primary outcome.

**Figure 2:**
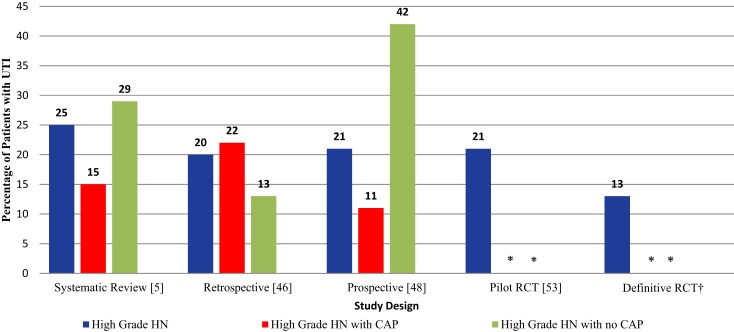
**UTI rates in patients with high-grade HN across different types of study design**. *Continuous antibiotic prophylaxis (CAP) use in hydronephrosis (HN) patients is unknown due to blinding. ^†^Ongoing trial.

**Figure 3:**
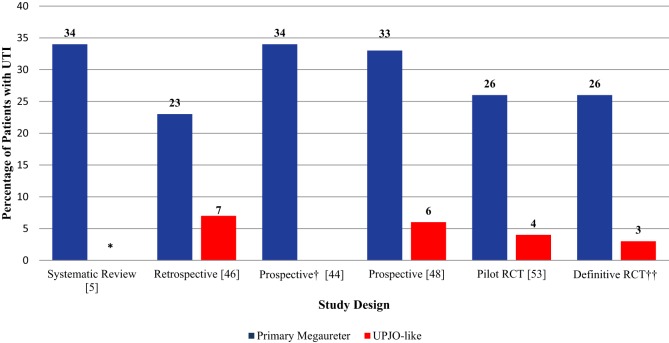
**UTI rates in patients with HN stratified by etiology (UPJO-like vs. primary megaureter)**. *Urinary tract infection rates in patients with UPJO-like was not reported due to paucity of data. ^†^Study only included patients with Primary Megaureter. ^‡^Ongoing trial.

The fUTI rate in the definitive RCT was substantially lower than the pooled UTI rate derived from our systematic review (13 vs. 25%, *p* < 0.05). Figure 2 displays UTI rates stratified by CAP use in patients with high-grade HN across different types of study design. Interestingly, 8/10 (80%) fUTIs in the definitive trial, occurred in uncircumcised boys as seen in Table 2. Similarly to our pilot data (49), patients with HUN had a sixfold higher UTI rate than those with isolated HN (Figure 3).

**Table 2:** **Characteristics of 10 participants with febrile UTIs**.

Patient – sex	Age (months)	HN side	HN grade	HN etiology	Ureteral diameter^a^ (mm)	Circumcision status	Surgery
1 – M	6.0	Bilat	III	HUN	10	No	No
2 – F	5.5	Bilat	IV	HUN	8	N/A	No
3 – M	7.0	Lt	III	UPJO	Not dilated	No	Pyeloplasty
4 – M	11.4	Lt	III	HUN	12	No	No
5 – M	8.1	Lt	IV	HUN	20	No	Ureterostomy and pyeloplasty
6 – M	6.3	Lt	III	HUN	10	No	No
7 – M	5.1	Lt	IV	UPJ + UVJ	10	No	Pyeloplasty and ureterocystostomy
8 – M	9.0	Lt	III	UPJO	Not dilated	No	No
9 – M	3.0	Rt	IV	HUN	14	No	No
10 – M	4.6	Rt	IV	HUN	15	Yes	Ureterocystostomy

*^a^Ureteral diameter was obtained from the ultrasound performed around the time of the febrile UTI*.

No side effects were associated with the use of the Trimethoprim according to the Data Safety Monitoring Board (DSMB) to date.

Of 78 patients who completed the study, only 1 (1.3%) was non-compliant (meaning that the child received the medication less than 75% of the time) based on information obtained from returned medication logs at follow-up clinic visits. Overall, three (3.8%) children missed one of the four required follow-up clinic visits. This high medication compliance and minimal loss to follow-up attested to the feasibility of this RCT.

## Future Directions

Despite controversial guidelines surrounding the management of prenatal HN, we have outlined the critical steps leading to a definitive multicenter RCT. While such studies are considered the gold standard in research, randomization alone does not warrant high methodological quality (55). Loss to follow-up can be a serious threat to the validity of studies. We, therefore, made it a priority to authenticate the feasibility of the ALPHA study through a pilot and interim descriptive analyses of the definitive study. Now that we have established true feasibility, our next step is to warrant a large sample size to ensure we obtain adequate power to properly answer the question regarding the efficacy of CAP in patients with prenatal HN. Large sample sizes are beneficial in that they enable investigators to detect small differences between treatments, minimizing findings due to chance (55). However, a single tertiary center simply does not have the necessary patient volume to complete the study in a timely manner (17% eligibility rate). For that reason, collaboration with multiple sites is key. Ultimately, the expansion of our collaborative network is driven by the need to provide meaningful data that guide clinicians and benefits patients.

A decline from 25 to 13% in the UTI rate of patients with high-grade HN was observed as the hierarchy of evidence progressed from observational studies to RCTs. This demonstrates that study designs lacking randomization, allocation concealment, or other strategies to minimize bias are more susceptible to overestimation of treatment effects. This finding highlights the importance of conducting properly powered, high-quality RCTs to answer clinically meaningful questions, such as the one regarding the effectiveness of CAP to prevent UTIs in infants with prenatal HN.

## Author Contributions

LB – design, analysis, and writing of the manuscript; BE – analysis of results and writing of the manuscript; KJ – analysis of results and writing of the manuscript; and AL – analysis, editing, and supervision of the manuscript.

## Conflict of Interest Statement

The authors declare that the research was conducted in the absence of any commercial or financial relationships that could be construed as a potential conflict of interest.
